# The BRG1 Chromatin Remodeler Protects Against Ovarian Cysts, Uterine Tumors, and Mammary Tumors in a Lineage-Specific Manner

**DOI:** 10.1371/journal.pone.0031346

**Published:** 2012-02-21

**Authors:** Daniel W. Serber, Allison Rogala, Maisam Makarem, Gary B. Rosson, Karl Simin, Virginia Godfrey, Terry Van Dyke, Connie J. Eaves, Scott J. Bultman

**Affiliations:** 1 Department of Genetics, University of North Carolina, Chapel Hill, North Carolina, United States of America; 2 Department of Pathology and Laboratory Medicine, University of North Carolina, Chapel Hill, North Carolina, United States of America; 3 Terry Fox Laboratory, British Columbia Cancer Agency, Vancouver, British Columbia, Canada; 4 Department of Medical Genetics, University of British Columbia, Vancouver, British Columbia, Canada; 5 Department of Medicine, University of British Columbia, Vancouver, British Columbia, Canada; 6 Department of Pathology and Laboratory Medicine, University of British Columbia, Vancouver, British Columbia, Canada; Massachusetts General Hospital, United States of America

## Abstract

The BRG1 catalytic subunit of SWI/SNF-related complexes is required for mammalian development as exemplified by the early embryonic lethality of *Brg1* null homozygous mice. BRG1 is also a tumor suppressor and, in mice, 10% of heterozygous (*Brg1^null/+^*) females develop mammary tumors. We now demonstrate that BRG1 mRNA and protein are expressed in both the luminal and basal cells of the mammary gland, raising the question of which lineage requires BRG1 to promote mammary homeostasis and prevent oncogenic transformation. To investigate this question, we utilized *Wap-Cre* to mutate both *Brg1* floxed alleles in the luminal cells of the mammary epithelium of pregnant mice where WAP is exclusively expressed within the mammary gland. Interestingly, we found that *Brg1^Wap-Cre^* conditional homozygotes lactated normally and did not develop mammary tumors even when they were maintained on a *Brm*-deficient background. However, *Brg1^Wap-Cre^* mutants did develop ovarian cysts and uterine tumors. Analysis of these latter tissues showed that both, like the mammary gland, contain cells that normally express *Brg1* and *Wap*. Thus, tumor formation in *Brg1* mutant mice appears to be confined to particular cell types that require BRG1 and also express *Wap*. Our results now show that such cells exist both in the ovary and the uterus but not in either the luminal or the basal compartments of the mammary gland. Taken together, these findings indicate that SWI/SNF-related complexes are dispensable in the luminal cells of the mammary gland and therefore argue against the notion that SWI/SNF-related complexes are essential for cell survival. These findings also suggest that the tumor-suppressor activity of BRG1 is restricted to the basal cells of the mammary gland and demonstrate that this function extends to other female reproductive organs, consistent with recent observations of recurrent *ARID1A/BAF250a* mutations in human ovarian and endometrial tumors.

## Introduction

Mammalian SWI/SNF chromatin-remodeling complexes regulate many cellular processes and function as tumor suppressors. Notably, the *BRG1*, *BRM*, *SNF5*, *ARID1A/BAF250a*, *PBRM1/BAF180*, and *Srg3/Baf155* subunits are consistently mutated or silenced in certain primary human tumors and also protect against tumorigenesis in mouse models [1]–[4]. Further evidence of the tumor-suppressor role of these genes has come from experiments showing that restoration of wild-type expression of the mutated or silenced subunit in tumor-derived cell lines can decrease proliferation and promote differentiation [5]. Mechanistically, several SWI/SNF subunits have been shown to physically interact with known tumor-suppressor genes and proto-oncogenes or their encoded proteins [1]–[3]. These studies include the demonstrated ability of the BRG1 catalytic subunit (also known as SMARCA4) and SNF5 (also known as BRG1-associated factor 47 or BAF47) to bind to the promoters of the p15^INK4b^ (known as p19 in the mouse), p16^INK4a^, and p21^CIP1/WAF1^ cyclin-dependent kinase (CDK) inhibitors and activate expression of these target genes [6]–[10]. This, in turn, leads to an inhibition of CDK2 or CDK4 and an accumulation of hypophosphorylated RB. BRG1 and an alternative catalytic subunit, BRM (also known as SMARCA2), can also bind to hypophosphorylated RB and are required to repress the activity of E2F1, inhibit the transcription of cyclins A and E, and mediate G_1_ cell-cycle arrest [11]–[15].

We previously showed that the homozygous *Brg1* null genotype is embryonic lethal in mice and 10% of *Brg1* null heterozygous mice spontaneously develop mammary tumors at approximately one year of age without prior exposure to ionizing radiation (IR) or other known oncogenic agents [16], [17]. These tumors do not show loss of heterozygosity (LOH) at the *Brg1* locus but do exhibit genomic instability suggesting that the acquisition of secondary mutations in addition to *Brg1* haploinsufficiency helps drive the development of the mammary tumors obtained. Interestingly, these *Brg1^null/+^* mammary tumors are more heterogeneous in terms of their histopathology, cytokeratin expression, and transcriptome profiles than the mammary tumors that arise in other mouse models of breast cancer [17]. To further investigate the role of *Brg1* as a tumor suppressor, we now report the results of experiments that demonstrate a relationship between the normal transcriptional activity of the *Brg1*, *Brm* and *Whey acidic protein* (*Wap*) genes, and the effect on viability and transformation of *Wap*-activated deletion of *Brg1* in the presence or absence of *Brm*.

## Results

### *Brg1* and *Brm* are co-expressed in all mammary epithelial cells, whereas *Wap* expression is confined to the luminal cells of the mammary gland in pregnant mice

**Figure 1:**
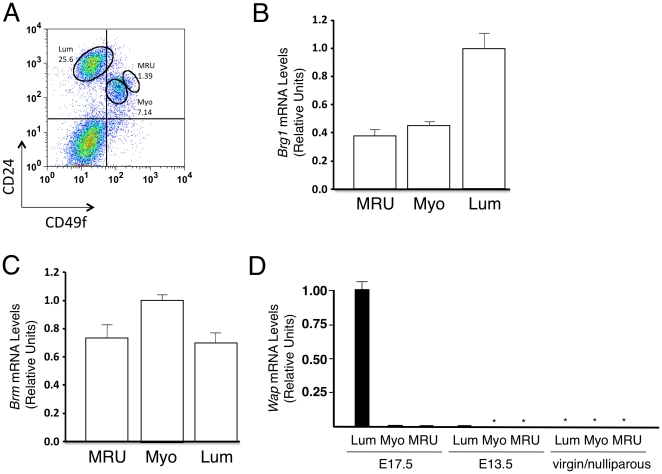
Expression of *Brg1*, *Brm*, and *Wap* in CD45^−^Ter119^−^CD31^−^ mammary gland subpopulations. A. Representative FACS plot of the subpopulations remaining after removing the hematopoietic and endothelial (CD45^+^Ter119^+^CD31^+^) cells from a suspension prepared from dissociated mammary tissue from a normal adult virgin mouse. The CD24^+^CD49f^low/−^ subset is enriched in luminal (Lum) cells; the CD24^+^CD49f^+^ subset is enriched in mature myoepithelial (Myo) cells; and the CD24^+^CD49^high^ subset is enriched in mammary stem cells (referred to as mammary repopulating units or MRU). B, C. RT-qPCR analysis of *Brg1* (B) and *Brm* (C) mRNA levels normalized to *Gapdh* levels in wild-type, flow-sorted mammary cell populations from adult virgin female mice. The Lum, Myo, and MRU subsets are as defined in panel A. Each histogram represents the mean ± SE from 3 independent experiments. D. RT-qPCR analysis of *Wap* mRNA levels normalized to *Gapdh* mRNA levels measured in the same 3 subsets. Lum, Myo, and MRU cells were isolated from pregnant females (E17.5 and E13.5) and virgin/nulliparous females (for representative FACS plots, see Figure 1). Asterisks indicate that the *Wap* signal was below the limit of detection. Each histogram represents the mean ± SE from 2 (E17.5) or 3 (E13.5, virgin/nulliparous) independent experiments.

In a first series of experiments, we sought to characterize the expression of *Brg1*, *Brm*, and *Wap* in different subsets of mammary cells in normal adult virgin and pregnant female mice. Accordingly, we dissociated their mammary gland fat pads into single-cell suspensions, removed hematopoietic, endothelial, and stromal cells, and subdivided the mammary epithelial cells into 3 fractions according to their levels of expression of CD24 and CD49f as described [18]. Representative fluorescent activated cell sorter (FACS) plots of the cells isolated for this analysis are shown in Figure 1A for cells from normal virgin mice and in Figure S1 for cells from pregnant mice. We then determined the levels of *Brg1*, *Brm* and *Wap* transcripts in each of these 3 subpopulations by RT-qPCR. As shown in Figure 1B, we found *Brg1* transcripts are present at readily detectable levels in all 3 fractions of normal adult virgin mice with the highest levels of expression in the CD24^+^CD49f^low/−^ (luminal cell-enriched) subset of cells and 2- to 3-fold lower levels in the CD24^+^CD49f^+^ and CD24^+^CD49f^high^ subsets, respectively. The latter 2 subpopulations are selectively enriched in mature myoepithelial cells and mammary stem cells (defined by their mammary repopulating activity in the cleared fat pad transplantation assay), respectively [18]. We found *Brm* is also expressed in all 3 of these same subpopulations (Figure 1C). This overlap in *Brg1* and *Brm* expression is similar to other adult tissues [19].

We then analyzed the same subsets of mammary cells for expression of the *Wap* gene. We found *Wap* is highly expressed in the CD24^+^CD49f^low/−^ (luminal) mammary cells during late pregnancy (E17.5) but not at an earlier stage (E13.5), nor in virgin/nulliparous females (Figure 1D). This is consistent with the known period of hormonally-induced *Wap* expression [20]. *Wap* transcripts were also not detectable in either of the other 2 subsets of basal mammary epithelial cells (Figure 1D). These findings predicted that a *Wap-Cre* transgene would delete *Brg1* only in the luminal cells of the mammary gland of mice and not until the mice had reached a late stage of pregnancy.

### Luminal mammary cells lacking *Brg1* remain viable and fully functional and do not generate tumors

**Figure 2:**
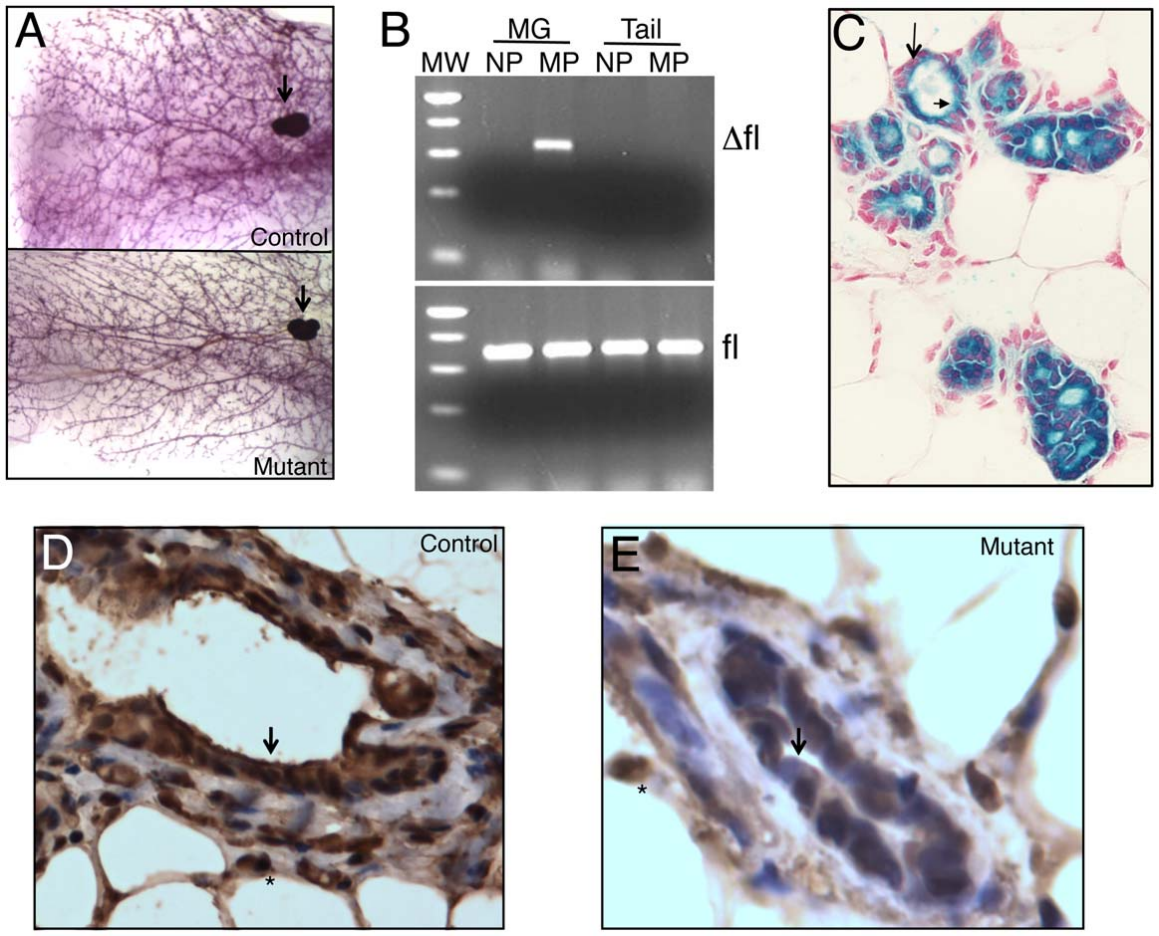
Lineage-specific deletion of *Brg1* in the mammary gland. A. Whole-mount preparation of control (top) and mutant (bottom) mammary glands from multiparous females. Asterisks, lymph nodes. B. Ethidium bromide-stained gels showing *Brg1* Δfl (top) and fl (bottom) PCR products. MW, molecular-weight standard (500-, 400-, 300-, 200-, and 75-bp fragments are visible); MG, mammary gland; NP, nulliparous; MP, multiparous. C. Mammary gland section from multiparous mouse carrying the *Wap-Cre* transgene on a *R26R* background. Cre activity, visualized as blue X-Gal staining, is restricted to luminal cells (arrowhead). Basal/myoepithelial cells (arrow) are negative and appear pink because of nuclear fast red counterstain. D, E. IHC staining of BRG1 showing strong staining in nuclei throughout the mammary gland in controls (D) but absent in luminal cells of mutant mice (E). Arrows, luminal cells; asterisks, adipocyte nuclei. 400× magnification.

To evaluate the function of BRG1 in the luminal lineage, we generated 21 female mice carrying a floxed *Brg1* gene and a *Wap-Cre* transgene [21], [22] (either as *Brg1^fl/fl^*:*Wap-Cre^+/0^* mice or as *Brg1^null/fl^*:*Wap-Cre^+/0^* mice, hereafter referred to as *Brg1^Wap-Cre^* mutants), and then monitored them for 15–19 months without exposure to IR or any other known oncogenic agents. To delete *Brg1* in the luminal cells, we mated all 21 females several times, starting at 2–3 months of age. Twelve littermates were monitored in parallel as controls. All of the females produced at least 2 litters each, and none showed any evidence of subsequent abnormalities. In particular, none of the mice developed any signs of mammary tumor formation (Table 1). Whole-mount preparations of their mammary glands removed after 15–19 months also did not reveal any evidence of microscopic tumors or altered morphology of their mammary glands (Figure 2A).

**Table 1:** Phenotype of *Brg1^Wap-Cre^* mutant mice.

				*Phenotype*	
*Gender*	*Category*	*Genotype* 1	*#Litters* *	*Mammary*	*Ovary/Uterus*
Male	control	+/floxed, Tg	N/A	none	N/A
Male	control^2^	null/floxed, Tg	N/A	none	N/A
Male	control^2^	null/floxed, Tg	N/A	none	N/A
Male	control^2^	floxed/floxed, Tg	N/A	none	N/A
Female	control	floxed/floxed	4	none	none^3^
Female	control	+/null, Tg	4	none	none
Female	control	+/+, Tg	4	none	none
Female	control	+/+, Tg	4	none	none
Female	control	+/floxed, Tg	2	none	none
Female	control	+/null	4	none	none
Female	control	null/floxed	3	none	none
Female	control	null/floxed	3	none	none
Female	control	null/floxed	3	none	none
Female	control	+/floxed, Tg	3	none	none
Female	control	null/floxed	2	none	none
Female	control	floxed/floxed	2	none	none
Female	mutant	null/floxed, Tg	4	none	uterine tumor
Female	mutant	null/floxed, Tg	4	none	ovarian cyst
Female	mutant	null/floxed, Tg	4	none	ovarian cyst, uterine tumor
Female	mutant	null/floxed, Tg	2	none	uterine tumor
Female	mutant	null/floxed, Tg	3	none	ovarian cyst
Female	mutant	null/floxed, Tg	3	none	none
Female	mutant	floxed/floxed, Tg	4	none	none
Female	mutant	floxed/floxed, Tg	3	none	ovarian cyst
Female	mutant	floxed/floxed, Tg	3	none	ovarian cyst, uterine tumor
Female	mutant	floxed/floxed, Tg	3	none	ovarian cyst
Female	mutant	floxed/floxed, Tg	3	none	ovarian cyst
Female	mutant	null/floxed, Tg	3	none	ovarian cyst
Female	mutant	floxed/floxed, Tg	2	none	ovarian cyst
Female	mutant	floxed/floxed, Tg	3	none	ovarian cyst
Female	mutant	floxed/floxed, Tg	3	none	ovarian cyst
Female	mutant	null/floxed, Tg	3	none	ovarian cyst
Female	mutant	floxed/floxed, Tg	3	none	ovarian cyst
Female	mutant	null/floxed, Tg	2	none	ovarian cyst
Female	mutant	null/floxed, Tg	2	none	ovarian cyst
Female	mutant	null/floxed, Tg	2	none	ovarian cyst
Female	mutant	floxed/floxed, Tg	2	none	none

*First pregnancy was at 2–3 months of age, and all mice were analyzed at 15–19 months of age.

1 Tg was hemizygous in each case (*Wap-Cre^+/0^*).

2 Mutant genotypically but considered a control because Cre transgene expression is induced by pregnancy, which does not occur in males.

3 Did not score for ovarian or uterine phenotype.

PCR assays of cells harvested from the mice at the end of the *Brg1^Δfl^* allele monitoring period confirmed that the *Brg1^fl^* allele had been converted to a recombined in mammary glands from multiparous but not nulliparous females (Figure 2B). Detection of the *fl* allele in the PCR assays was not unexpected because mammary epithelial cells are embedded in a fat pad that also contains lymph nodes and blood vessels, and *Wap-Cre* is not expressed in adipocytes, lymphocytes, or other stromal cells such as fibroblasts. The *Brg1^Δfl^* PCR product also could not be detected in tail tissue from either multiparous or nulliparous females (Figure 2B) consistent with the target specificity of action of the *Wap-Cre* transgene [22]–[24].

To further characterize the mammary cells in which *Brg1* is deleted in *Brg1^Wap-Cre^* mutants, we introduced the Rosa26 reporter (*R26R*) gene into the *Brg1^Wap-Cre^* mice [25]. X-Gal staining of tissue removed from multiparous females that were not at that time either pregnant, lactating, or involuting confirmed that the *Wap*-activated Cre activity had been restricted to the luminal cells of the mammary epithelium (Figure 2C). Next, we performed immunohistochemical (IHC) studies of sections of mammary tissue from multiparous *Brg1^Wap-Cre^* mutants and control nulliparous mice. These experiments showed that the usually strong and widespread nuclear staining for BRG1 protein (Figure 2D and Figure S2A) was diminished or absent in the luminal cells of the conditional mutants while persisting in stromal cells of the same animals where *Wap-Cre* is not expressed and *Brg1* was not mutated (Figure 2E). Taken together, these results demonstrate that *Brg1* is mutated in the expected luminal lineage cell-specific manner and argues strongly against a defect in Cre-mediated recombination as an explanation for the lack of mammary tumors in *Brg1^Wap-Cre^* mutant mice.

### Mammary luminal cells lacking *Brg1* do not undergo aberrant apoptosis

**Figure 3:**
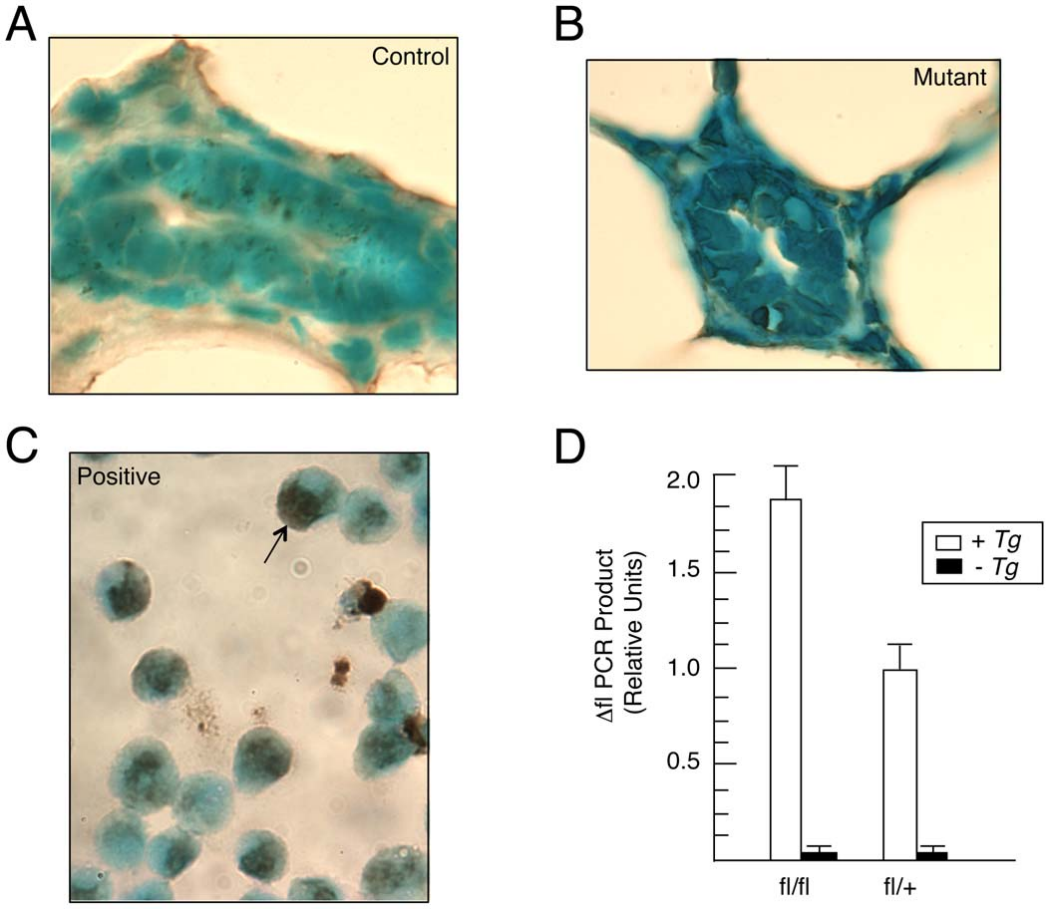
Mutant luminal cells do not undergo aberrant apoptosis. A, B. TUNEL assay of control (A) and mutant (B) adult mammary gland sections counterstained with methyl green. C. Lymphocytes induced to undergo apoptosis have brown nuclei (arrow) and serve as a positive control. D. qPCR analysis of *Brg1* Δfl genomic DNA levels normalized to *Gapdh* genomic DNA levels in mammary glands. Each histogram represents the mean ± SE from 3 independent experiments.

It was reported that a heterozygous deletion of *Brg1* in certain lung cells, makes them prone to urethane-induced tumorigenesis, whereas a homozygous deletion of *Brg1* in the same cells does not because the homozygous deletion induces apoptosis [26]. To investigate whether aberrant apoptosis (unrelated to involution) might account for the lack of mammary tumors in *Brg1^Wap-Cre^* mutant mice, we performed TUNEL assays on mammary glands harvested from multiparous females that were not pregnant, lactating, or involuting at the time. In controls, we observed minimal apoptosis as only 0.25% of the mammary epithelial cells were TUNEL-positive (Figure 3A), which is consistent with previous reports of wild-type mammary glands [27]. And in contrast to the lung tumor study, we did not detect a significant difference in the frequency of apoptosis in mutants, where only 0.20% of the cells were TUNEL positive (Figure 3B). In these experiments, a leukemia cell line induced to undergo apoptosis served as a positive control for the TUNEL staining (Figure 3C).

We also performed qPCR analyses to determine whether *Brg1*-deleted cells are significantly underrepresented when the abundance of the Δfl PCR product in mammary gland tissue from conditional homozygous (*Brg1^fl/fl^*:*Wap-Cre^+/0^*) and conditional heterozygous (*Brg1^fl/+^*:*Wap-Cre^+/0^*) multiparous females was compared. Unlike the lung cancer study, which reported a 1∶8 ratio of Δfl PCR product in homozygous versus heterozygous mice [26], we obtained a ∼2∶1 ratio of Δfl PCR product in a comparison of our homozygous and heterozygous mice, which was what was expected if the Δfl/Δfl cells were not being strongly selected against by activation of either apoptosis or necrosis mechanisms (Figure 3D).

Finally, we noted that every *Brg1^Wap-Cre^* mutant female that became pregnant not only delivered a healthy litter but was also able to lactate and raise normal-sized pups. This finding indicates that the alveolar cell proliferation and differentiation that underlies mammary gland development during pregnancy and lactation had not been impaired in spite of the lack of *Brg1* expression. A significant increase in apoptosis would have resulted in insufficient milk production and neonatal runting or lethality of their progeny as observed for mammary-specific mutations of other genes [28]–[33]. Thus, overall we could not find any evidence of decreased viability of *Brg1*-deficient luminal cells.

### *Brm* is not required to compensate for the loss of *Brg1* in luminal mammary cells

BRG1 and BRM are 75% identical, broadly expressed, and serve as alternative catalytic subunits of SWI/SNF-related complexes with similar or identical activities [34]–[35]. To determine whether the lack of a phenotype in *Brg1*-deleted luminal mammary cells might be explained by functional compensation from the co-expressed *Brm*, we transferred the *Brg1^Wap-Cre^* mutation onto a *Brm*-deficient background. We then monitored 8 double-mutant females and 6 control (non-transgenic) females for 15–19 months without exposure of either to IR or any other known oncogenic agents (Table 2). To inactivate *Brg1* in each of the 14 females, a first pregnancy was initiated at 2–3 months of age, and then again 1–2 times subsequently. From each of these mice, 2–3 litters of normal sized pups were obtained, indicating that the luminal cells of the mammary gland can remain viable and completely functional even when neither *Brg1* nor *Brm* is present. Nor did any of these mice develop mammary tumors (Table 2). These results demonstrate that the “normal” phenotype of *Brg1* null luminal cells is not due to a compensatory activity being provided by *Brm* nor are either of these catalytic subunits required to prevent these cells from generating tumors.

**Table 2:** Phenotype of *Brg1^Wap-Cre^* mutant mice on a *Brm*-deficient background.

				*Phenotype*	
*Gender*	*Category*	*Genotype* 1	*#Litters* *	*Mammary*	*Ovary/Uterus*
Female	control	floxed/floxed, Brm^−/−^	3	none	none
Female	control	floxed/floxed, Brm^−/^	3	none	none
Female	control	floxed/floxed, Brm^−/−^	3	none	none
Female	control	floxed/floxed, Brm^−/−^	2	none	none
Female	control	floxed/floxed, Brm^+/−^	2	none	none
Female	control	floxed/floxed, Brm^−/−^	3	none	none
Female	double mutant	floxed/floxed, Tg, Brm^−/−^	3	none	none
Female	double mutant	floxed/floxed, Tg, Brm^−/−^	3	none	ovarian cyst
Female	double mutant	floxed/floxed, Tg, Brm^−/−^	2	none	ovarian cyst
Female	double mutant	floxed/floxed, Tg, Brm^−/−^	2	none	none
Female	double mutant	floxed/floxed, Tg, Brm^−/−^	3	none	ovarian cyst
Female	double mutant	floxed/floxed, Tg, Brm^−/−^	2	none	ovarian cyst
Female	double mutant	floxed/floxed, Tg, Brm^−/−^	3	none	none
Female	double mutant	floxed/floxed, Tg, Brm^−/−^	2	none	ovarian cyst

*First pregnancy was at 2–3 months of age, no lactation defects were observed, and all mice were analyzed at 15–19 months of age.

1 Tg was hemizygous in each case (*Wap-Cre^+/0^*).

### Mammary tumor induction caused by inhibition of the RB pathway is not altered by *Wap-Cre*-mediated deletion of *Brg1* in luminal cells

BRG1 interacts with RB and is required for RB-mediated growth arrest in tumor-derived cell lines *in vitro* [11]–[15]. However, it is not clear whether this mechanism applies to cancer prevention *in vivo*. In a previous study, we noted that the induction of *Rb^+/−^* mammary tumors was not altered on a *Brg1^null/+^* background [17]. To determine whether this would also extend to *Brg1* null luminal cells, we crossed *Wap-T121* transgenic mice with our *Brg1^Wap-Cre^* mutants and then monitored them for tumor formation. T121 contains the first 121 amino acids of the SV40 large T antigen, which binds to RB as well as the other 2 pocket proteins (p107 and p130) and perturbs their function [32]. *Wap-T121* mice express this transgene in their mammary luminal cells and develop aggressive mammary tumors with 100% penetrance by ∼16 months of age [32]. We confirmed this finding but observed no further change in the penetrance or latency (Figure S3) or the histopathologic characteristics (data not shown) of the tumor phenotype in 10 *Wap-T121*, *Brg1^Wap-Cre^* double mutant mice as compared to mice expressing only *Wap-T121*.

### *Wap-Cre*-mediated deletion of *Brg1* induces ovarian cysts and uterine tumor formation

**Figure 4:**
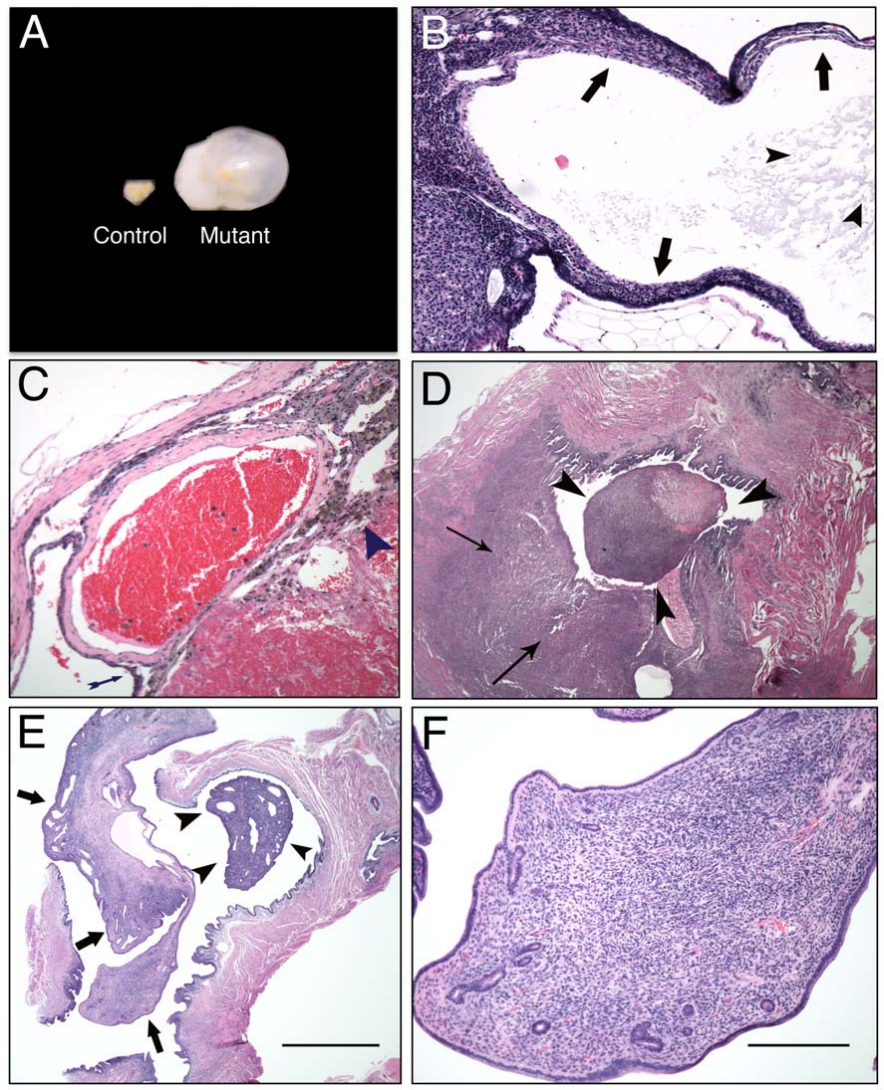
Histopathology of ovarian cysts and uterine tumors from *Brg1^Wap-Cre^* mice. A. Control ovary (left) and cystic ovary from a conditional *Brg1^Wap-Cre^* mutant (right). B. Section of an ovarian cyst stained with H&E at 40× magnification. The thin wall of this multilocular structure is comprised of an inner layer of spindle-shaped cells surrounded by multiple layers of round cells with uniformly sized, round, basophilic nuclei with scant cytoplasm (arrows). This cyst is filled with a lightly eosinophilic proteinaceous fluid (arrowheads). C. Hemorrhagic ovarian cyst section stained with H&E at 40× magnification. The thin arrow points to a wall of the cyst, and the arrowhead points to a region of hemosiderin-laden macrophages at the edge of the hemorrhage. D. Histiocytic sarcoma section stained with H&E at 20× magnification. A pleomorphic population of cells with basophilic round to ovoid nuclei and cytoplasm varying from scant to abundant and foamy are seen infiltrating the myometrium (thin arrows) and forming a polyp (arrowheads) projecting into the uterine lumen E. Endometrial stromal polyp section stained with H&E at 20× magnification. The polyp (arrows) includes a pedunculated mass (arrowheads) that projects into the uterine lumen. F. Endometrial stromal polyp section stained with H&E stained at 200× magnification. The polyp stroma is comprised of spindle cells with variable amounts of cytoplasm and ovoid nuclei; the structure also includes small blood vessels and small endometrial glands and is covered by a single layered cuboidal to columnar epithelium with basally located round nuclei.

Although *Brg1^Wap-Cre^* multiparous females are not predisposed to develop breast cancer, we discovered that they do develop grossly visible ovarian cysts and uterine neoplasms in contrast to age-matched sibling control females in none of whom such pathologies occurred (Table 1). The ovarian cyst phenotype was highly (76%) penetrant, affecting 16 out of 21 conditional-mutant females. The cystic ovaries ranged in size from 5–12 mm (Figure 4A) and were unilateral in 12/16 females and bilateral in 4/16 females. The affected ovaries contained 1–3 cysts, which is less than the number present in polycystic ovaries [36]. In addition, *Brg1^Wap-Cre^* mutant females did not exhibit hirsutism or reduced fertility as young adults over several litters, which is characteristic of human polycystic ovarian disease caused by altered hormonal influences such as hyperandrogenism [37]. We also did not detect a significant difference in circulating progesterone levels (Figure S4), which might have provided insight into the polycystic ovaries or the lack of mammary tumors in *Brg1^Wap-Cre^* mutants.

Histopathological analysis of H&E-stained ovary sections revealed follicular cysts lined with either spindle cells or columnar cells characteristic of having apical ciliary structures (Figure 4B). All cysts contained either serous fluid or varying stages of hemorrhage ranging from acute to chronic based upon the degree of organization (Figure 4B, C). Serous cysts were encapsulated by an inner layer of spindle-shaped cells, a middle layer of granulosa cells, and an outer layer of spindle cells (Figure 4C). The uterine neoplasm phenotype was 19% penetrant, occurring in 4 of 21 conditional mutant females (Table 1). These neoplasms were of 2 histopathologic types: either histiocytic sarcomas projecting into the uterine lumen (Figure 4D) or endometrial stromal polyps (Figure 4E, F).

IHC analysis demonstrated that BRG1 is normally expressed in both the ovary and the uterus. In the ovary, BRG1 protein was detected in oocytes, granulosa cells, and theca cells within the follicles (Figure S2B). In the uterus, BRG1 was expressed in a widespread manner (Figure S2C). In the experiments where we performed X-Gal staining on *R26R:Wap-Cre^+/0^* tissues, Cre activity was detected consistently in the ovary and uterus as well as in the mammary glands of 10 *Wap-Cre* transgenic mice but not in negative controls carrying *R26R* but lacking the *Wap-Cre* transgene. These findings are consistent with endogenous *Wap* expression in the ovary and uterus [37]. Ovarian Cre activity was observed in granuolosa cells within follicles (Figure S5). Similarly, BRG1 immunostaining was abolished or diminished in these cells in the *Brg1^Wap-Cre^* mutants (Figure S2D, E). The results presented above strongly suggest that the generation of the ovarian cysts and uterine tumors is a cell autonomous process.

## Discussion

We previously demonstrated that *Brg1^null/+^* mice are susceptible to mammary tumorigenesis, but the cells of origin were not identified [16], [17]. We now show that this is unlikely to be a consequence of decreased *Brg1* expression in luminal cells as mammary tumors were never obtained in *Brg1^Wap-Cre^* conditional mutants in spite of the fact that *Brg1* is normally expressed in these cells and was successfully deleted by forcing the mice to undergo multiple pregnancies but without any evidence of increased apoptosis. These findings imply that *Brg1* haploisufficiency must activate an oncogenic process in other cells, either members of the basal mammary cell compartment and/or stromal cells. Consistent with this hypothesis is our finding that *Brg1* is normally expressed in the basal cells of the mammary gland but the *Brg1* floxed allele could not be deleted in *Brg1^Wap-Cre^* conditional mutant mice because expression of *Wap* and hence *Cre* is not induced in the basal cells. Additional support for an important role of *Brg1* in mammary stem cells is the demonstration of its requirement for embryonic stem (ES) cell self-renewal and pluripotency [38], [39], as well as other, more restricted, types of stem cells [40], [41]. A *Brg1* stem/progenitor cell-restricted function in the mammary gland is also consistent with the diverse histopathological characteristics and transcriptome profiles of *Brg1^null/+^* tumors [17]. It may also explain why depletion of BRG1 from MCF-10A mammary cells, which have characteristics of non-malignant luminal cells, did not increase their proliferative activity nor confer a tumor-like phenotype [42].

The restricted expression of *Wap-Cre* to the luminal cells in the mammary gland may explain why only certain *Wap-Cre* conditional mutants develop highly penetrant tumor phenotypes. For example, *Wap-Cre* driven mutation of *Smad4* and *Brca2* causes a very high frequency of affected mice to develop mammary tumors (100% and 77%, respectively), whereas only 15% of mice with *Wap-Cre* driven mutation of *Brca1* develop mammary tumors and no tumors are obtained following *Wap-Cre* driven mutation of *Pparγ* or *Stat3* [43]–[47]. Failure of tumorigenesis would be expected if the gene being targeted for deletion was not expressed in a luminal cell, or not required, or involved in a pathway whose perturbation would lead to deregulated growth of a luminal cell. In addition, *Wap-Cre* driven mutations that require other genetic or epigenetic changes to be accumulated might not lead to tumors because of the transient lifespan of the luminal compartment.

SWI/SNF-related complexes are essential for the development of many cell lineages [48], which suggests that they might be required for the viability of most or all primary cell types. In fact, the only cells previously known to deficient for both BRG1 and BRM are certain tumor-derived cell lines [2], [5], and these tumor cells may have subverted the normal requirement for at least one catalytic subunit *via* inhibition of apoptosis. However, our present findings now offer a potential alternative explanation; i.e., that SWI/SNF-related complexes may be dispensable in mammary luminal cells. Here we show that neither *Brg1* nor *Brm* are required for a morphologically- and functionally-normal mammary gland and their absence did not affect the ability of the gland to support the repeated production of litters of normal-sized pups. Thus, SWI/SNF-related complexes catalyzed by either BRG1 or BRM must be dispensable for the viability and normal functionality of mammary luminal cells, in spite of previous experiments with EpH4 cells expressing a dominant-negative BRG1 suggesting that casein expression is dependent on SWI/SNF catalytic activity [49]. It is likely that other primary cell types can also survive in the absence of BRG1/BRM-catalyzed SWI/SNF complexes, and we have evidence from *Villin-Cre* experiments that this is the case for intestinal epithelial cells (data not shown).

Although *Brg1^Wap-Cre^* mice did not develop mammary tumors, they did become susceptible to the formation of ovarian cysts and uterine tumors. The ovarian cysts were associated with a loss of BRG1 in granulosa cells (Figure S5), whereas our previous analysis of *Brg1^Zp3-Cre^* conditional mutants indicated that ovarian cysts did not develop when *Brg1* was deleted in developing oocytes [50]. These results are compatible with ovarian cysts arising from functional defects in somatic support cells rather than germ cells. BRG1 could prevent cyst formation in wild-type cells through its well-known role in development and differentiation [2], [48]. Alternatively, it could be promoting apoptosis to promote the death of immature ovarian follicles (i.e., artesia) [36]. The uterine neoplasms are also noteworthy because few genetically engineered mouse models of uterine cancer other than *Pten* have been described [51]–[53]. The *Brg1^Wap-Cre^* ovarian cyst and uterine tumor phenotype is also reminiscent of recent deep-sequencing efforts demonstrating consistent mutations of another SWI/SNF subunit, *ARID1A/BAF250a*, in ∼30% and ∼50% of human ovarian clear cell carcinomas and endometrial carcinomas, respectively [54], [55]. Our data also support the observation that BRG1 is downregulated in human cervical carcinomas [56]. In summary, our results add weight to the idea that SWI/SNF-related complexes have an important function in preventing the development of cancers, particularly within the stem/progenitor compartments of cells in certain tissues.

## Materials and Methods

### Ethics statement, mice, and genotyping

All mouse experiments were approved by the Institutional Animal Care and Use Committees (IACUC) review board at the University of North Carolina as approved protocol ID #10-026 and were performed in accordance with federal guidelines. *Wap-Cre* transgenic mice were obtained from the Jackson Laboratory (Bar Harbor, ME, USA). The *Brg1* floxed and Δfloxed alleles were genotyped by PCR as previously described [21]. Quantification of the relative abundance of the *Brg1* Δfloxed allele was performed by qPCR and normalized to *Gapdh* as previously described [26].

### Histology

Normal tissues and cystic or tumor tissues were fixed in 4% paraformaldehyde, embedded in paraffin, and 5 µm sections were cut according to standard procedures. Sections were either stained with H&E or processed for IHC using a BRG1 rabbit polyclonal antibody (Upstate/Millipore #07-478, Temecula, CA, USA) or for TUNEL assays (Chemicon/Millipore, Temecula, CA, USA) according to the manufacturer's recommendations. The TUNEL positive control was provided by the manufacture and consisted of human promyelocytic leukemia treated with actinomycin D. Whole mount preparations of mammary glands and X-Gal staining of R26R (*Rosa-lox-stop-lox-LacZ*) tissues were performed following standard procedures. Serum progesterone levels were determined by ELISA.

### Isolation of mammary cell populations

Mammary glands were dissected from 8–12 week old, female C57BL/6J mice before or after induction of pregnancy (E13.5 or E17.5) as indicated. Single-cell suspensions were generated and analyzed by flow cytometry as previously described with minor modifications [18]. Briefly, mammary glands were digested overnight at 37°C in DMEM/F12 medium containing 1 mg/mL collagenase A (Roche) and 100 U/mL hyaluronidase (Sigma). After vortexing and lysis of red blood cells in NH_4_Cl, the nucleated cells were further dissociated in 0.25% trypsin, 5 mg/mL dispase with 0.1 mg/mL DNase I (Sigma), and filtered through a 40 µm mesh to remove cell clumps and aggregates. Cells were then treated with anti-CD16/CD32 Fcγ III/II receptor antibody (BD Pharmingen) and subsequently with anti-CD45 (Biolegend), Ter119 (Biolegend), CD31(BD Pharmingen)-biotinylated antibodies followed by streptavidin-phycoerythrin (SA-PE, BD Pharmingen) and with fluorescein isothiocyanate (FITC)-conjugated anti-CD49f (clone GoH3, BD Pharmingen) and allophycoerythrin (APC)-conjugated anti-CD24 (clone M1/69, Biolegend) to isolate the fractions indicated on a FACSDiva or Influx (BD) fluorescence-activated cell sorter (FACS).

### RT-qPCR

RNA from mammary cell fractions was isolated using Absolutely RNA nano and microprep kits (Stratagene, La Jolla, CA, USA) and reverse transcribed using SuperScript III RT (Invitrogen) according to the manufacturer's protocol. Validated TaqMan assays (Applied Biosystems, Foster City, CA, USA) were used with TaqMan gene expression master mix (Applied Biosystems) on an ABI 7300 instrument under default cycling conditions (95°C 15 s followed by 60°C 1 min for 45 cycles). *Gapdh* was used as a normalization control. Relative expression levels were determined using ΔΔCt method. Control reactions lacking RT yielded little or no signal.

## Supporting Information

Figure S1 **Representative FACS plots showing the expression of CD24 and CD49f at various developmental stages after the depletion of hematopoietic and endothelial cells (CD45^+^CD31^+^Ter119^+^).** Mammary cells were isolated from adult virgin (left) and pregnant mice at E13.5 (middle), and E17.5 (right). CD24^+^CD49f^low/−^, CD24^+^CD49f^+^, and CD24^+^CD49f^high^ populations were isolated as shown above.
(TIF)

Figure S2 **BRG1 IHC of mouse tissues.** (A) Wild-type mammary gland section at 1000× magnification showing strong nuclear expression in mammary epithelial cells (luminal cells in particular) and stromal cells. (B) Wild-type ovarian section at 400× magnification showing strong staining throughout follicle including oocyte, granulosa and theca cells. (C) Wild-type uterine section at 1000× magnification showing widespread expression. (D, E) Expression in granulosa cells within ovarian follicle is high in wild-type mice (D) and markedly diminished in conditional mutant mice (E).
(TIF)

Figure S3 ***Wap-Cre*** **-mediated deletion of** ***Brg1*** **does not exacerbate the** ***Wap-T121*** **phenotype.** Kaplan-Meier survival curve showing the fraction of palpable mammary tumor free mice.
(TIF)

Figure S4 **Serum progesterone levels from controls (** ***Brg1^fl/fl^*** **) and mutants (** ***Brg1^fl/fl^:Wap-Cre^+/0^*** **) mice while non-pregnant and pregnant (at E17.5).** Histograms represent mean ± SE for 3 independent experiments.
(TIF)

Figure S5 **Ovary section from mouse carrying** ***Wap-Cre*** **transgene and** ***R26R*** **reporter following X-Gal staining.** Blue Cre-positive cells are present in granulosa cells within the follicle.
(TIF)
