# Supplementary figures and images for: The BRG1 Chromatin Remodeler Protects Against Ovarian Cysts, Uterine Tumors, and Mammary Tumors in a Lineage-Specific Manner
Source: PLoS One. 2012 Feb 21;7(2):e31346. doi: 10.1371/journal.pone.0031346 (PMC3283619; doi:10.1371/journal.pone.0031346)

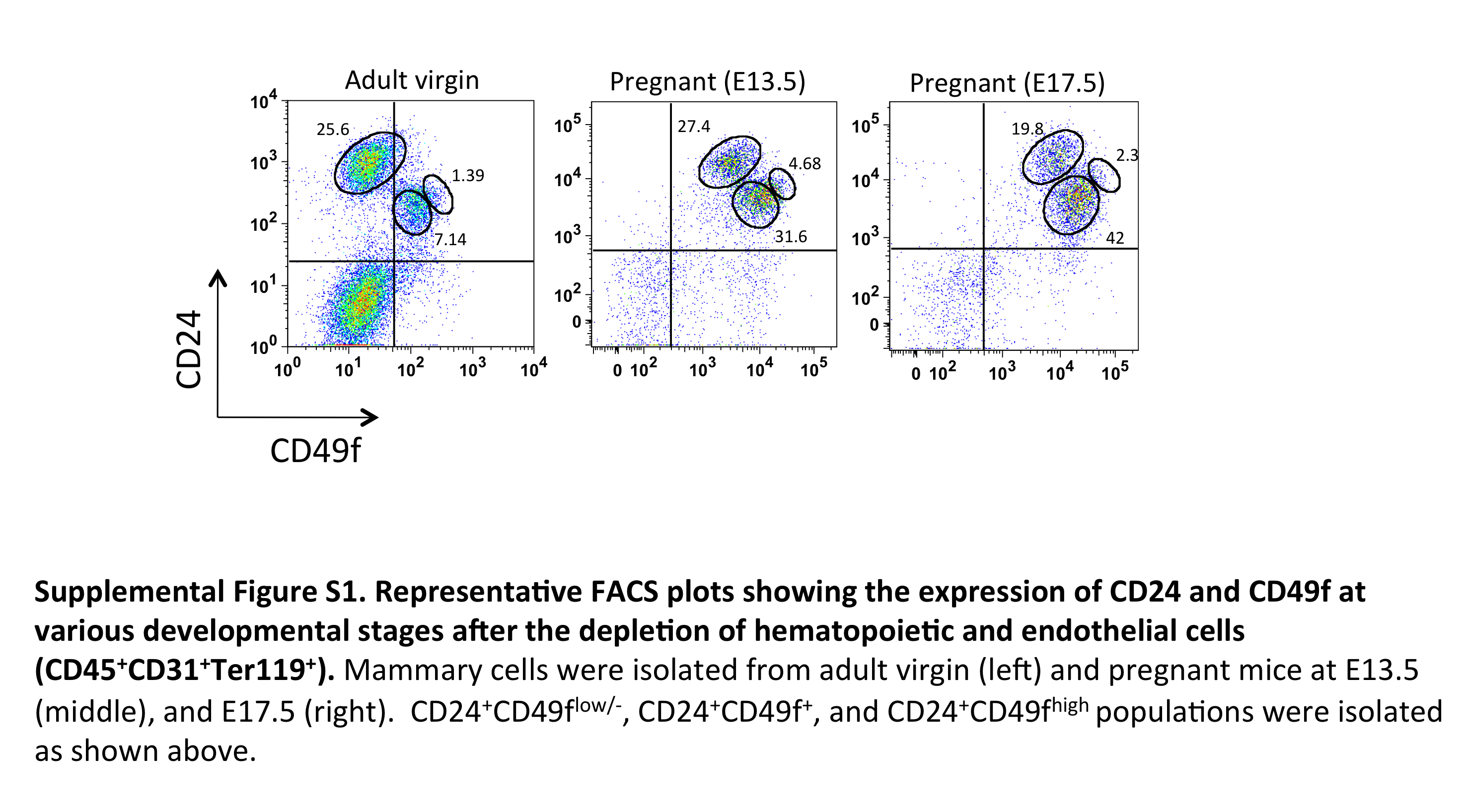

Supplement: Figure S1 — Representative FACS plots showing the expression of CD24 and CD49f at various developmental stages after the depletion of hematopoietic and endothelial cells (CD45+CD31+Ter119+). Mammary cells were isolated from adult virgin (left) and pregnant mice at E13.5 (middle), and E17.5 (right). CD24+CD49flow/−, CD24+CD49f+, and CD24+CD49fhigh populations were isolated as shown above. (TIF) [file pone.0031346.s001.tif]

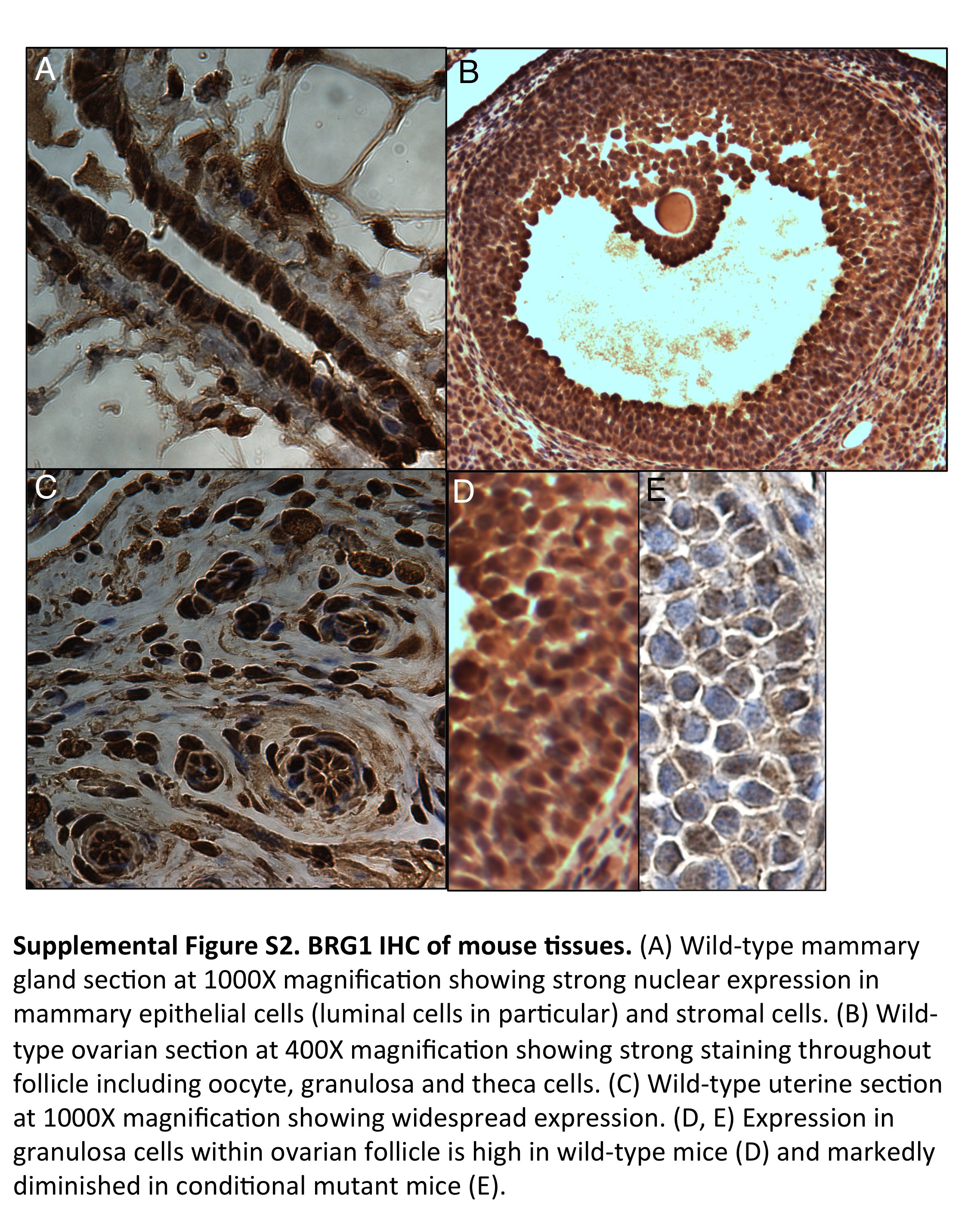

Supplement: Figure S2 — BRG1 IHC of mouse tissues. (A) Wild-type mammary gland section at 1000× magnification showing strong nuclear expression in mammary epithelial cells (luminal cells in particular) and stromal cells. (B) Wild-type ovarian section at 400× magnification showing strong staining throughout follicle including oocyte, granulosa and theca cells. (C) Wild-type uterine section at 1000× magnification showing widespread expression. (D, E) Expression in granulosa cells within ovarian follicle is high in wild-type mice (D) and markedly diminished in conditional mutant mice (E). (TIF) [file pone.0031346.s002.tif]

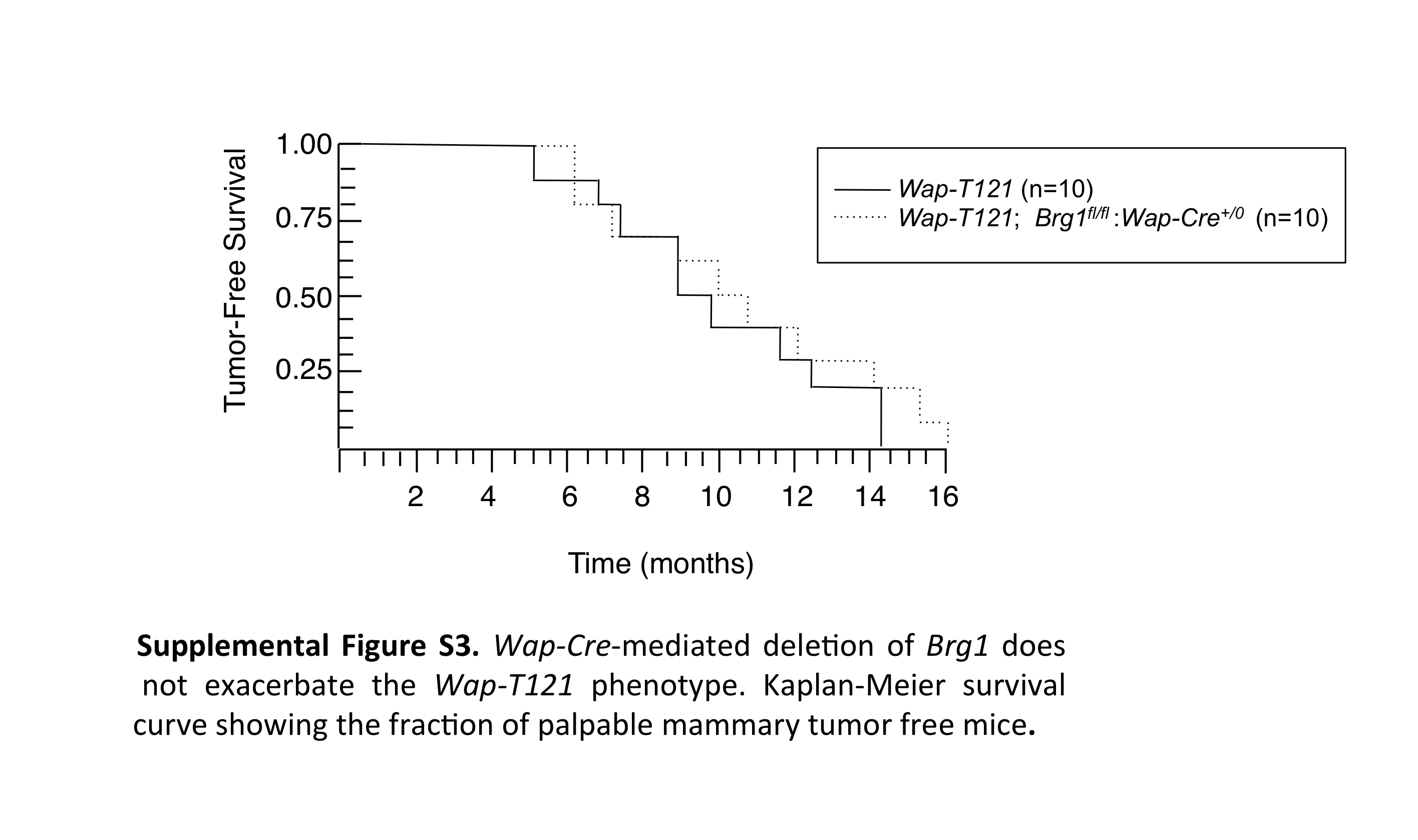

Supplement: Figure S3 — Wap-Cre -mediated deletion of Brg1 does not exacerbate the Wap-T121 phenotype. Kaplan-Meier survival curve showing the fraction of palpable mammary tumor free mice. (TIF) [file pone.0031346.s003.tif]

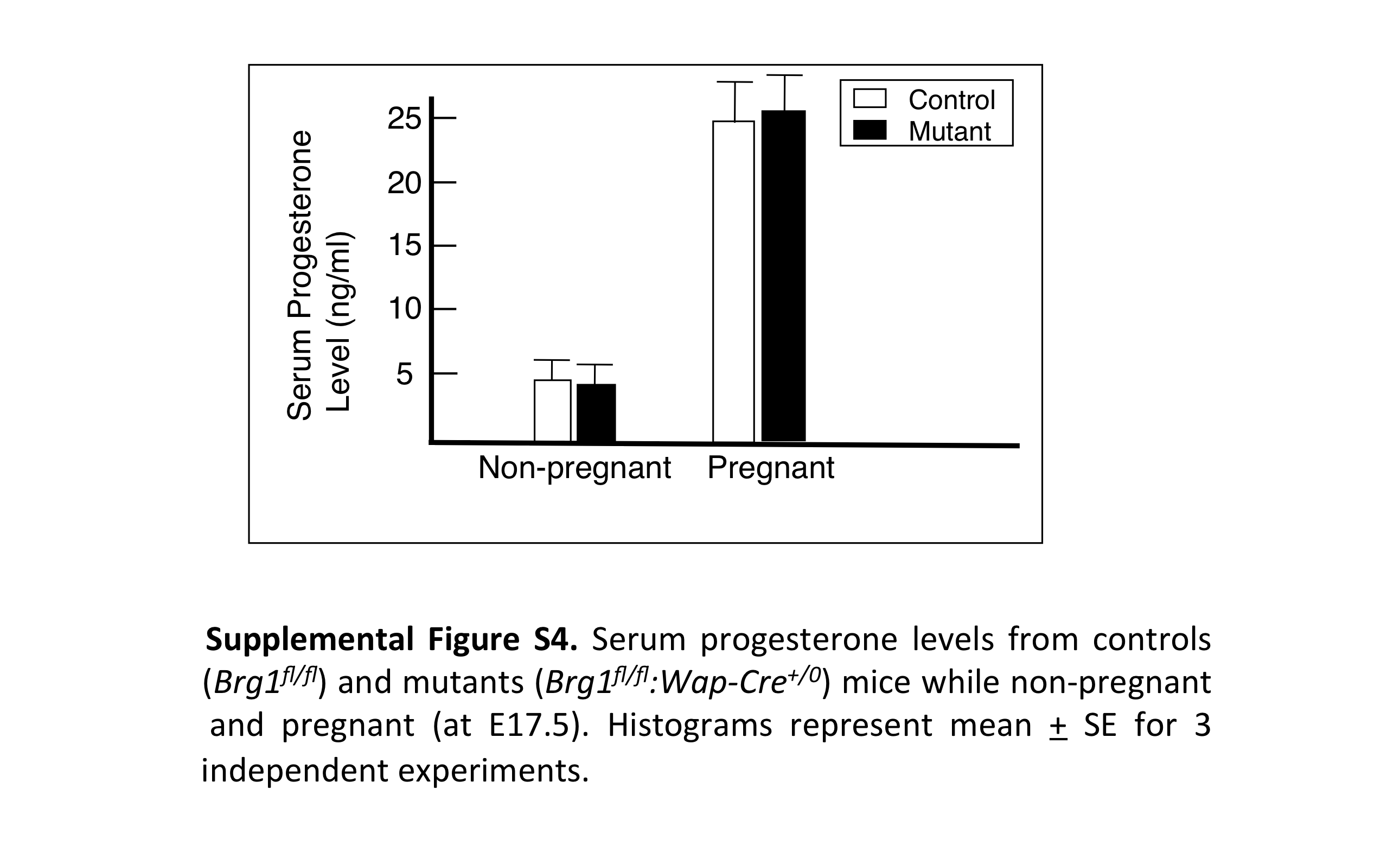

Supplement: Figure S4 — Serum progesterone levels from controls ( Brg1fl/fl ) and mutants ( Brg1fl/fl:Wap-Cre+/0 ) mice while non-pregnant and pregnant (at E17.5). Histograms represent mean ± SE for 3 independent experiments. (TIF) [file pone.0031346.s004.tif]

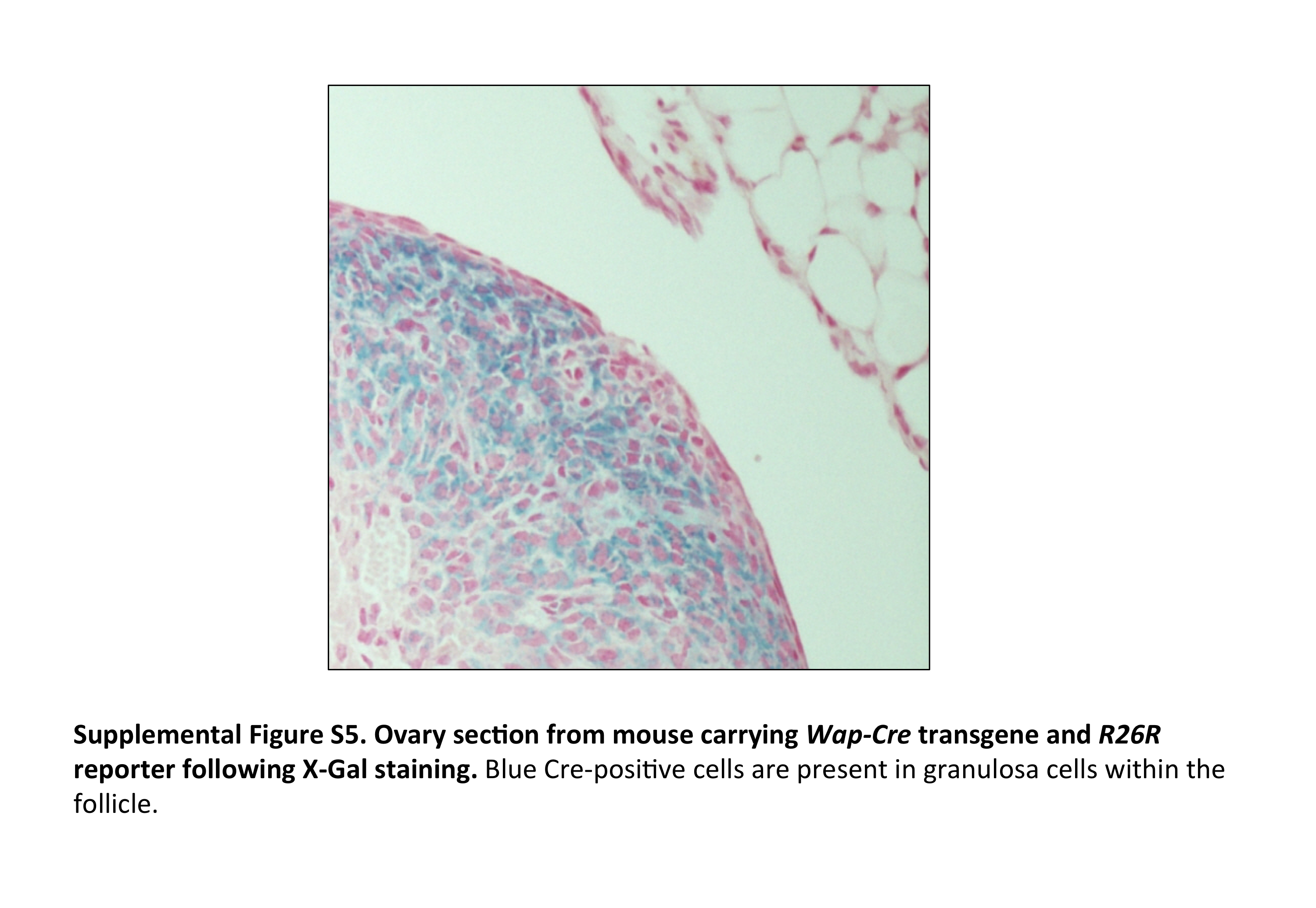

Supplement: Figure S5 — Ovary section from mouse carrying Wap-Cre transgene and R26R reporter following X-Gal staining. Blue Cre-positive cells are present in granulosa cells within the follicle. (TIF) [file pone.0031346.s005.tif]
